# Silicon and Iron Differently Alleviate Copper Toxicity in Cucumber Leaves

**DOI:** 10.3390/plants8120554

**Published:** 2019-11-28

**Authors:** Dragana Bosnić, Predrag Bosnić, Dragana Nikolić, Miroslav Nikolić, Jelena Samardžić

**Affiliations:** 1 Laboratory for Plant Molecular Biology, Institute of Molecular Genetics and Genetic Engineering, University of Belgrade, Vojvode Stepe 444a, 11000 Belgrade, Serbia; dragana.bosnic@imgge.bg.ac.rs (D.B.); dragana.nikolic.imgge@rcub.bg.ac.rs (D.N.); 2 Department of Plant Nutrition, Institute for Multidisciplinary Research, University of Belgrade, Kneza Višeslava 1, 11000 Belgrade, Serbia; predrag.bosnic@imsi.bg.ac.rs (P.B.); mnikolic@imsi.bg.ac.rs (M.N.)

**Keywords:** copper toxicity, micronutrient deficiency, silicon, iron, nicotianamine, histidine, Cu-chelation

## Abstract

Copper (Cu) toxicity in plants may lead to iron (Fe), zinc (Zn) and manganese (Mn) deficiencies. Here, we investigated the effect of Si and Fe supply on the concentrations of micronutrients and metal-chelating amino acids nicotianamine (NA) and histidine (His) in leaves of cucumber plants exposed to Cu in excess. Cucumber (*Cucumis sativus* L.) was treated with 10 µM Cu, and additional 100 µM Fe or/and 1.5 mM Si for five days. High Cu and decreased Zn, Fe and Mn concentrations were found in Cu treatment. Additional Fe supply had a more pronounced effect in decreasing Cu accumulation and improving the molar ratio between micronutrients as compared to the Si supply. However, the simultaneous supply of Fe and Si was the most effective treatment in alleviation of Cu-induced deficiency of Fe, Zn and Mn. Additional Fe supply increased the His but not NA concentration, while Si supply significantly increased both NA and His whereby the NA:Cu and His:Cu molar ratios exceeded the control values indicating that Si recruits Cu-chelation to achieve Cu tolerance. In conclusion, Si-mediated alleviation of Cu toxicity was directed toward Cu tolerance while Fe-alleviative effect was due to a dramatic decrease in Cu accumulation.

## 1. Introduction

Micronutrients such as copper (Cu), iron (Fe), zinc (Zn) and manganese (Mn), are essential to all plants, since they participate in numerous metabolic processes. The interaction between the elements becomes apparent when the excessive level of one of the micronutrients in the growth medium is present so that it may interfere with the uptake, transport and accumulation of the other nutrients, consequently disturbing the overall plant nutrient balance [1]. Copper present at high concentration has phytotoxic effects such as inhibition of plant growth and development, and generation of oxidative stress [2]. Plants exposed to overload of Cu are highly prone to the induced deficiency of other essential ions thus suffering a disturbance of essential metabolic processes [1]. Being a transition metal, Cu in excess competes for specific binding sites of other metal ions and consequently displace them from their binding sites [2]. Most of the plant metal transporters have limited ion specificity, therefore there are no efficient mechanisms for plants to differentially regulate the uptake of specific metal ion and avoid competition [3]. Divalent cation uptake transporters such as: Iron-regulated transporter 1 (IRT1), Zrt/IRT-like protein (ZIP) transporter family, natural resistance-associated macrophage proteins (NRAMPs) are able to unselectively mediate the transport of a broad range of metals (Fe, Zn, Cu, Mn and Cd) depending on their availability [3,4,5]. The copper and Fe competition is of particular concern since they are redox active counterparts and their metabolic crossroads are in tight relationships [6]. It has been proposed that high Cu induces alterations in Fe nutrition by decreasing the root Fe availability and Fe acquisition processes along with inhibition of Fe-deficiency responses [7,8,9,10]. Excess Cu competes with Fe for the reduction sites, i.e., Fe (III) reductase (FRO) activity, which precedes the uptake of both metals [11]. Accordingly, inhibition of root FRO activity with increased Cu supply has been found in cucumber [7,12]. However, it has also been noticed that additional Fe supply can be used for mitigation of the deleterious effects caused by high Cu and for correcting Fe deficiency induced by excess of Cu [13,14,15].

Silicon (Si) is not essential for most of the plant species; however, its presence in plants may influence plant response to both mineral toxicity and deficiency stress [16,17]. For example, addition of Si to Cu-stressed cucumber plants enhances accumulation of Cu-binding molecules (proteins and organic acids) that could buffer excess Cu, thus diminishing the level of oxidative stress [18]. However, there are only a few studies addressing nutrient imbalances in plant leaves under Cu toxicity and Si nutrition. Lower leaf Cu and increased calcium (Ca) concentration was found to be one of the main beneficial effects of Si nutrition for both *Zinnia elegans* and *Erica andevalensis* exposed to toxic Cu [19,20]. This was attributed to the well-known antagonistic effect of Cu and Ca, which was alleviated by Si supplementation. In addition, Si may alleviate the excess of Cu by increasing the uptake of other essential metals such as Mn and Zn [21].

Apart from the metal uptake, the tissue metal partition and distribution may also be affected by metal imbalances. Nicotianamine (NA) is a non-proteinogenic amino acid and a major metal chelator involved in the homeostasis of metals such as Cu, Fe, Zn and Mn. NA is essential for metal distribution in leaves via phloem and seed loading of Cu, Fe, Zn and Mn [22,23,24]. However, NA has an exclusive role in Cu metabolism, since it is also responsible for xylem transport of Cu but not of other metals [25,26], and contrary to Fe, Mn- and Zn-, the Cu-NA complex is very stable at mild acidic pH [27,28]. NA was suggested to participate in heavy metal stress [28]. Moreover, binding of metals by strong ligands such as NA is considered as the main detoxification strategy in non-hypertolerant plants [29].

Besides NA, the amino acid histidine (His) is another important metal-chelator. This amino acid is critical for metal coordination to metalloproteins and metal binding to the active site of metalloenzymes [30]. Free His was found to be implicated in metal hypertolerance [31,32] in addition to its decisive role in metal hyperaccumulation as shown for Ni [33]. Both NA and His form complexes with Cu with stability constants much higher than for other elements [34,35]. 

This study investigates for the first time how Si and/or Fe nutrition modulates the leaf micronutrient status under Cu toxicity stress, with special emphasis on the distinctive responses of metal-chelating amino acids (NA and His).

## 2. Results

### 2.1. Plant Growth and Symptoms of Copper Toxicity Stress

Total dry biomass of hydroponically grown cucumber plants was the lowest in Cu treatment (Table 1, Figure S1). Cu + Si treatment increased plant biomass by 32% as compared to Cu treatment. There was no difference in the total plant biomass between Cu + Fe and Cu + Fe + Si treatments and the control cucumber plants (Table 1). In comparison with the control, a significant decrease in the leaf DW was only recorded in Cu treatment (Table 1).

### 2.2. Concentration and Content of Microelements (Cu, Fe, Zn and Mn) in Cucumber Leaves

**Figure 1:**
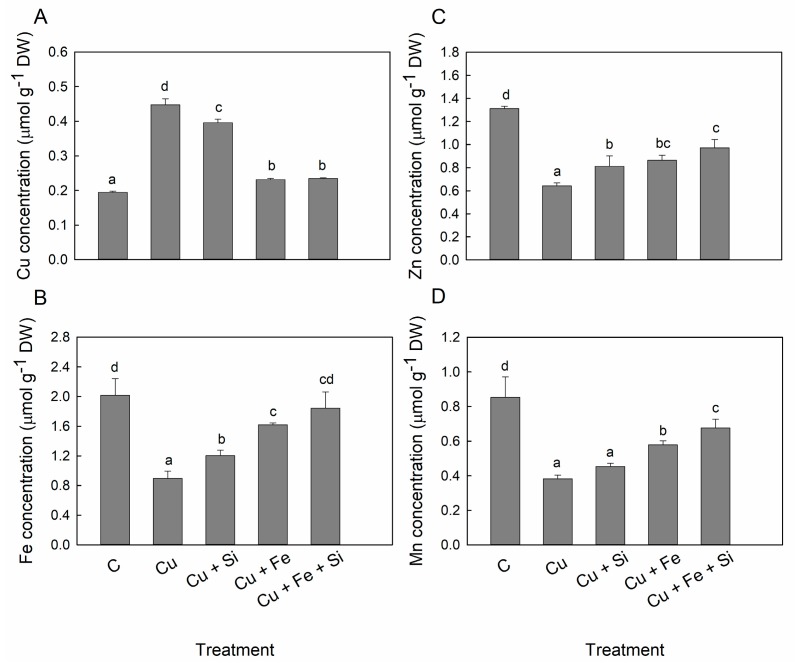
Concentration of Cu (**A**), Fe (**B**), Zn (**C**) and Mn (**D**) in cucumber leaves. Plants were harvested after 5 days of treatment with excess Cu (Cu), excess Cu and Si supply (Cu + Si), excess Cu and additional Fe supply (Cu + Fe) or combined excess Cu and additional Fe and Si supply (Cu + Fe + Si); control plants (C) remained untreated. Bars are means of four replicates ± SD. Different letters indicate significant differences among the treatments at *p* < 0.05.

Copper treatment resulted in a 2.3-fold increase in the Cu concentration in the leaves (Figure 1A). Si supply to the Cu treatment slightly but significantly (*p* < 0.05) decreased the Cu concentration (Figure 1A). However, there was no difference in leaf Cu content between Cu and Cu + Si treatments due to the changes in leaf dry weight (Table 2). Both Cu + Fe and Cu + Fe + Si treatments decreased remarkably the Cu concentration and content almost two-fold in comparison to only Cu-treated plants (Figure 1A, Table 2). The leaf concentrations of Zn, Fe and Mn were decreased about 2-fold in Cu treatment, compared to the control and it gradually increased in Cu + Si, Cu + Fe and Cu + Fe + Si treatment, respectively (Figure 1B–D). The most effective improvement in the microelement concentration was observed for Fe in Cu + Fe + Si treatment, reaching the control values (Figure 1B). The leaf content of Fe, Zn and Mn was also calculated and presented in Table 2. A remarkable decrease in the content of all examined elements, particularly for Mn (three-fold) was found in Cu treatment. Treatments: Cu + Si, Cu + Fe and Cu + Fe + Si gradually increased the content of Fe, Mn and Zn, reaching up to 87%, 71% and 63%, of the control level in Cu + Fe + Si treatment, respectively (Table 2).

### 2.3. Molar Ratio between Micronutrients

The molar ratio between elements was calculated and presented in Table 3. The lowest Fe:Cu, Zn:Cu and Mn:Cu molar ratios were noticed in Cu treatment; the Fe:Cu ratio was five-fold lower in comparison to control, whereas the Zn:Cu ratio was seven-fold decreased, from 7:1 to 1:1. The Mn:Cu molar ratio in Cu treatment was decreased to 1:1, as well. However, Cu + Si treatment increased the Fe:Cu (3:1) and Zn:Cu (2:1) ratios, while Mn:Cu ratio was the same as in Cu treatment. The Fe:Cu molar ratio was significantly improved in both Cu + Fe and Cu + Fe + Si treatments, 7:1 and 8:1, respectively (Table 3). The Zn:Cu ratio was the same in those two treatments, 4:1, while higher than in other treatments. Unlike Cu and Cu + Si, the Cu + Fe and Cu + Fe + Si treatments improved the Mn:Cu ratio, to 2:1 and 3:1, respectively (Table 3).

### 2.4. Concentration of NA and the Molar Ratio of NA to Cu, Fe, Zn and Mn

**Figure 2:**
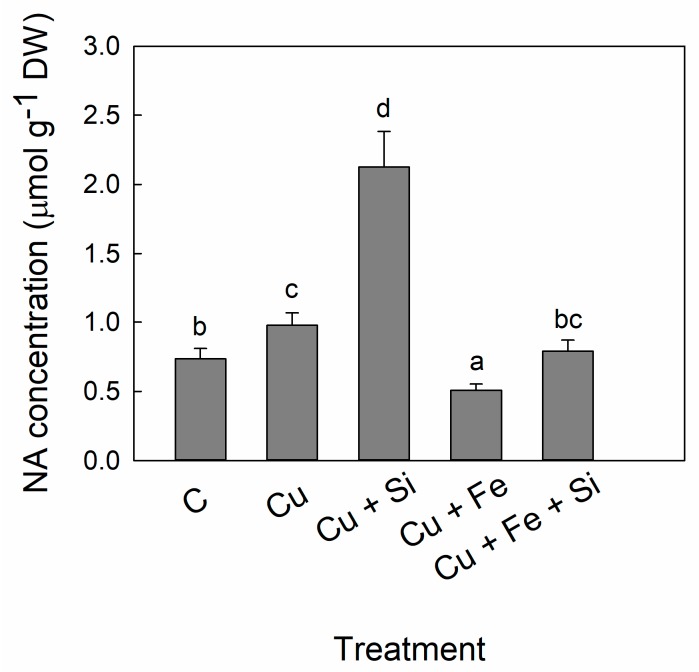
Concentration of nicotianamine (NA) in cucumber leaves. Plants were harvested after 5 days of treatment with excess Cu (Cu), excess Cu and Si supply (Cu + Si), excess Cu and additional Fe supply (Cu + Fe) or combined excess Cu and additional Fe and Si supply (Cu + Fe + Si); control plants (C) remained untreated. Bars are means of four replicates ± SD. Different letters indicate significant differences among the treatments at *p* < 0.05.

NA accumulation in leaves was induced in Cu treatment by 30% compared to control (Figure 2). Cu + Si treatment had the highest increase in NA concentration, about 300% of the control values. In Cu + Fe plants NA concentration was decreased to 70% of the control plants values. NA concentration in Cu + Fe + Si treatment was slightly higher compared to that in the control plants though not statistically significant (Figure 2).

The molar ratio of NA to microelements in leaves of control cucumber plants was found to be as following: 3.8:1, 0.4:1, 0.6:1 and 0.9:1 for Cu, Fe, Zn and Mn, respectively. The molar ratio NA:Cu was decreased in Cu treatment while the molar ratios of NA to other elements (Fe, Zn and Mn) were significantly increased compared to control (Table 4). In Cu + Si treatment, the NA:Cu ratio was the highest among all treatments, exceeding the control value, and this treatment resulted in additionally increased the ratio between NA and the other elements compared to Cu treatment. On the contrary, the NA:Cu ratio was decreased in Cu + Fe treatment while combined Cu + Fe + Si treatment restored the NA:Cu ratio almost to the control level (Table 4). The NA:Fe, NA:Zn and NA:Mn molar ratios in Cu + Fe and Cu + Fe + Si treatments were similar to the control values (Table 4). 

### 2.5. Concentration of His and the Molar Ratio of His to Cu, Fe, Zn and Mn

**Figure 3:**
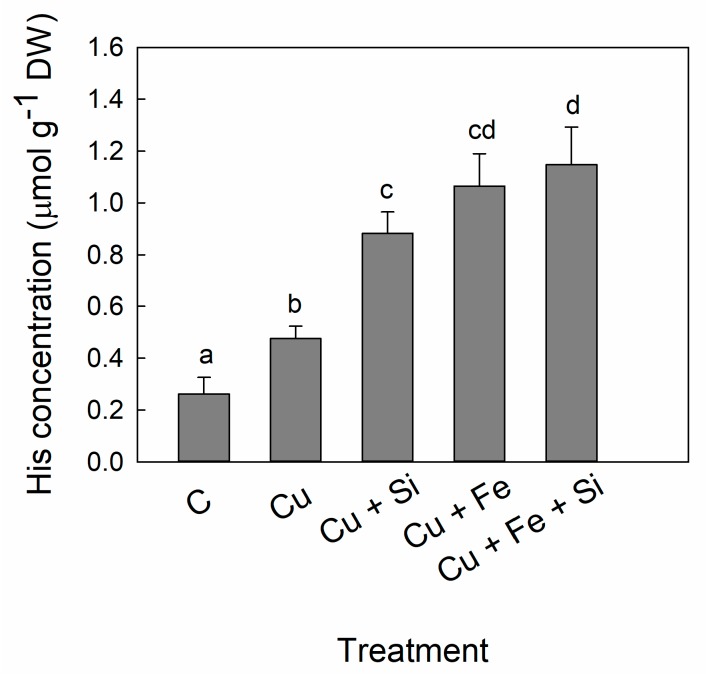
Concentration of histidine (His) in cucumber leaves. Plants were harvested after 5 days of treatment with excess Cu (Cu), excess Cu and Si supply (Cu + Si), excess Cu and additional Fe supply (Cu + Fe) or combined excess Cu and additional Fe and Si supply (Cu + Fe + Si); control plants (C) remained untreated. Bars are means of four replicates ± SD. Different letters indicate significant differences among the treatments at *p* < 0.05.

A continuous increase in His concentration was found in all treatments starting from Cu (1.8-fold) to Cu + Fe + Si treatment (5.4-fold) compared to control values (Figure 3).

The changes in His and metal concentrations consequently altered the His:metal molar ratios (Table 5). In Cu treatment the His:Cu molar ratio was decreased compared to control, while Cu + Si treatment increased it above the control level. Both Cu + Fe and Cu + Fe + Si treatments had many-fold higher the His:Cu molar ratio then the control. In accordance with total His concentration, the molar ratios: His:Fe, His:Zn and His:Mn were several times greater in all examined treatments: Cu, Cu + Si, Cu + Fe and Cu + Fe + Si (Table 5).

## 3. Discussion

The primary site of Cu toxicity is the root system; therefore many studies have been devoted to investigation of the detrimental effects of excess Cu in roots [36,37,38]. In our previous study, we have shown the toxic effects of 10 µM Cu treatment to cucumber plants where copper was highly accumulated in the root, reaching more than 10-fold higher values than in the leaf [18]. Apart from high Cu accumulation, excess of Cu also causes unbalanced uptake of other mineral nutrients [1,10]. Deficiency of both macro- and micronutrients was found in the leaves of different plant species exposed to Cu stress [39,40]. Herein we focused on the fine changes in molar ratios between Cu and other micronutrients and on the role of chelating amino acids in leaf under ameliorative effect of Si and/or additional Fe.

Decreased concentration and content of Fe, Zn and Mn were recorded in the leaves of Cu-treated cucumber along with higher Cu accumulation (Figure 1, Table 2). It is well documented that addition of Si may improve the nutritional status of plants despite nutrient imbalances in the growth medium [16]. Cucumber is one of the few Si-accumulating dicots with a relatively high capacity for Si accumulation, which has been shown in numerous studies [16]. In our previous study we have found that Si supply decreased Cu uptake in cucumber plants by enhancing the Cu deposition in the root cell wall of the Cu-treated cucumber [18]. However, in studies dealing with Fe, Zn or Mn deficiency the beneficial effect of Si was attributed to the metal remobilization and enhanced metal distribution rather than to a direct effect on the metal uptake [41,42,43,44,45]. In the present work, Si supply decreased Cu accumulation; however, there was no difference in Cu content between Cu and Cu + Si treatments due to the biomass increase of Cu + Si plants (Figure 1, Table 1 and Table 2). Additionally, Si supply increased Fe, Zn and Mn concentrations and contents (Figure 1, Table 1). Similarly, in *Erica andevalensis* leaf Si supply significantly increased Zn concentration, which was lowered due to the competitive Cu–Zn interaction [19]. Si addition also restored the leaf Mn concentration in *Spartina densiflora* subjected to Cu stress [46]. Moreover, it was observed in the current study that the molar ratios of Fe:Cu and Zn:Cu were improved in the Si-supplied cucumber plants (Table 3). The optimal micronutrient ratios in plants are considered just as important as their absolute concentration particularly in terms of induced deficiency of specific nutrient [47]. Taken together, our results indicate that Si supply can alleviate to some extent the antagonistic effect of excess Cu on Fe, Zn and as well as the Mn accumulation in the leaves.

Interestingly, additional Fe supply was more effective in preventing Cu accumulation in cucumber leaves than Si supply (Figure 1A, Table 2). It has been reported that addition of Fe could ameliorate Cu toxicity symptoms due to the antagonistic interaction between Cu and Fe [7,40]. Studies in bean and maize have shown that additional Fe mitigates Cu accumulation by outcompeting Cu uptake, thus improving plant growth and decreasing oxidative stress [13,14]. We have also found that application of Fe to Cu-stressed plants resulted in a greater increase of Fe, Zn and Mn concentrations and contents than addition of Si, thus significantly improving the Fe:Cu and also Zn:Cu and Mn:Cu molar ratios, which were disturbed by the Cu treatment (Figure 1B–D, Table 3). The molar ratio between elements is in some cases the decisive indicator of stress; for example the molar ratio of Fe to other elements, rather than the total Fe concentration, has been found to be crucial for the initiation of Fe-deficiency response [48].

Simultaneous supply of Si and Fe had no additional effect regarding the Cu concentration in the leaves (Figure 1). On the other hand, this was the most effective treatment in respect to the increasing contents of Zn and Mn (Table 2). Moreover, this treatment restored the Fe content to the control level (Table 2). Consequently, the Fe:Cu, Zn:Cu and Mn:Cu molar ratios in the Cu + Fe + Si treatment were closest to the control values among all the treatments studied (Table 3). Therefore, we suggest that only when simultaneously applied with Si, the additional Fe supply could reach its full ameliorative potential regarding alleviation of the microelement deficiency induced by excess of Cu.

The accumulation of free amino acids is considered as an active response of plants to heavy metal stress [49]. Indeed, it has been shown that plants respond to excess of Cu by accumulating free amino acids [50]. However, the amount of certain amino acids rises rather than others, it refers primarily to NA and His, which have the highest binding constants for Cu compared to those for other metal ions [34,35]. Cu-NA and Cu-His complexes have been detected in plant tissues in different plant species [51,52,53]. In the present study, we recorded increased NA and His accumulation in the cucumber leaves under Cu toxicity stress (Figure 2 and Figure 3); however, the NA:Cu and His:Cu molar ratios decreased compared to those in the control plants (Table 4 and Table 5). Similar to our results, in the xylem sap from chicory and tomato plants both the NA and His concentrations were found elevated by Cu treatment [50]. Pich and Scholz [25] suggested that the two-fold higher NA concentration found in the leaves of Cu-treated tomato was required for the protection against phytotoxicity caused by excess Cu.

Interestingly, Si supply to Cu-stressed cucumber plants led to an additional increase in concentration of both amino acids (Figure 2 and Figure 3). The study with Cu-treated bamboo revealed a high proportion of amino groups involved in Cu complexation, along with an increased His accumulation in Si-supplied plants [52]. This could be specific for stress conditions; however, there are no reports about Si influence on amino acids in non-stressed plants so far.

In the present study, the NA:Cu and His:Cu molar ratios were significantly improved in Cu + Si treatment so that they even exceeded the control values (Table 4 and Table 5). The molar ratio of NA to metals was found constant during the plant development and as such is important for ensuring efficient loading and transport of essential micronutrients [23]. However, the significant surplus of metal chelators indicated by the molar ratio, could be decisive for achieving the tolerance to metal toxicity. The higher NA:Cu and His:Cu molar ratios, exceeding the control values, in the Si-supplied plants suggest that Si recruits the Cu-chelators to attain higher Cu tolerance. Chelation is considered as a potential mechanism that governs metal tolerance in plants by controlling the concentration of the free metal ions [54]. We have already reported that the decreased oxidative stress in the Cu + Si-treated plants as well as their improved tolerance to Cu are attributable to Cu-buffering by the increased levels of Cu-binding proteins and organic acids [18]. Taken together, the Cu-binding and Cu-chelating compounds are important mechanism of Si-alleviative effect in Cu toxicity stress.

Apart from Cu, NA is indispensable in homeostasis of Fe, but its role is ambiguous in Fe deficiency. It has been reported that the Fe deficiency did not cause biosynthesis and accumulation of NA in dicots [26,55,56]. Surprisingly, both overproduction and low NA resulted in decreased availability of Fe and affected Fe deficiency response [57,58]. Therefore, it is indicative that an optimal NA:Fe ratio is necessary for the Fe homeostasis so that an imbalance of this ratio, resulting from high or low NA, would lead to the Fe deficiency response. However, it has been found that addition of Si stimulated the NA production in Fe-deficient cucumber, leading to enhanced remobilization of Fe from the older to younger cucumber leaves [59]. Therefore, NA may have an important role in the Si-stimulated metal remobilization during metal deficiency and metal buffering in metal toxicity.

Additional Fe supply did not increase NA concentration, which is in accordance with the strong decrease of Cu accumulation observed in Cu + Fe treatment (Figure 2). However, simultaneous application of Fe and Si to Cu-stressed plants increased NA concentration, which resulted in an improved NA:Cu molar ratio, reaching the control level (Table 3). Interesting results have been obtained for His in the Cu + Fe and Cu + Fe + Si treatments. Although we recorded a lower Cu concentration in leaf compared to the concentration in Cu treatment, increased His levels were found in both Cu + Fe and Cu + Fe + Si treatments (Figure 3). Contrary to our results, concentration of His in the xylem sap of *Brassica carinata* followed the increasing trend proportional to the rising Cu concentration and had the greatest relative increase among all the amino acids analyzed [60]. Surprisingly, in the same study NA was not induced under Cu excess but rather under Cu deficiency although NA had the highest absolute concentration compared to all amino acids analyzed [60]. As suggested by Irtelli et al. [60], response to Cu stress is not only species-specific but it is also stress-specific.

## 4. Materials and Methods 

### 4.1. Plant Material and Growth Conditions

Seeds of cucumber (*Cucumis sativus* L. cv. Chinese long) were surface sterilized with sodium hypochlorite, soaked in a saturated calcium sulfate solution overnight and then transferred for germination on moist filter papers for 4 days in the dark at 25 °C. The seedlings were grown for 12 days in preculture nutrient solution containing (in mM): 0.7 K_2_SO_4_, 0.1 KCl, 2.0 Ca (NO_3_)_2_, 0.5 MgSO_4_ and 0.1 KH_2_PO_4_, and (in µM): 0.5 MnSO_4_, 0.5 ZnSO_4_, 0.2 CuSO_4_, 0.01 (NH_4_)_6_Mo_7_O_24_, 10 H_3_BO_3_ and 20 Fe^III^EDTA. Nutrient solution was constantly aerated and renewed completely every two days. pH was adjusted to 6 and daily checked. Plants were grown at 24/20 °C (light/dark) with a day/night regime of 16/8 h and 250 μmol photons m^−2^ s^−1^ light intensity at plant height (provided by led panels; Apollo 8, Cidly Co., Ltd., Shenzhen, China). The experimental set up included five different groups of plants: control plants grown in standard solution containing 0.2 μM Cu and 20 μM Fe (C); plants treated with 10 μM Cu supplied as CuSO_4_ into solution, which contained 20 μM Fe (Cu treatment); plants grown in standard solution with 20 μM Fe and treated with 10 μM Cu and 1.5 mM Si (Cu + Si treatment); plants treated with 10 μM Cu and 100 μM Fe (Cu + Fe treatment) and the last treatment contained combined 10 μM Cu, 100 μM Fe and 1.5 mM Si (Cu + Fe + Si treatment). Fe was supplied as Fe^III^EDTA and Si was supplied as Si (OH)_4_ freshly prepared by passing Na_2_SiO_3_ through a cation-exchange resin (Amberlite IR-120, H^+^ form; Acros Organics, Geel, Belgium); The experiments were performed in four replicates (pots) with four plants in each pot. 

### 4.2. Visual Observation, Plant Harvesting and Biomass Recording

The visual symptoms were recorded and the plants were harvested five days after the onset of treatments. One subsample was oven-dried at 70 °C for 72 h, weighed and subsequently ground for microelement determination. The samples for amino acids analysis were instantly frozen in liquid nitrogen after harvesting.

### 4.3. Determination of Microelements Cu, Fe, Zn and Mn

Dry leaf material was ground and digested in a mixture of 3 mL concentrated HNO_3_ and 2 mL H_2_O_2_ in a microwave oven (Speedwave MWS-3+; Berghof Products + Instruments GmbH, Eningen, Germany). Cu, Fe, Zn and Mn concentrations were determined by inductively coupled plasma optical emission spectroscopy (ICP-OES, Spectro-Genesis EOP II; Spectro Analytical Instruments GmbH, Kleve, Germany). The analytical accuracy of total concentrations was evaluated using certified reference material (GBW 10015 Spinach, Institute of Geophysical and Geochemical Exploration, Langfang, China).

### 4.4. Extraction and Analysis of Amino Acids Nicotianamine and Histidine

For extraction of nicotianamine, leaf tissue was homogenized in liquid nitrogen and extracted in ddH_2_O, incubated at 80 °C for 30 min and after centrifugation for 10 min at 16,000× *g* the supernatant was filtered and used for HPLC analysis, similarly as in Mendoza-Cózatl et al. [61]. For histidine determination, leaf tissue was homogenized in liquid nitrogen and extracted in 50% methanol, centrifuged at 4 °C and the filtered supernatant was used for analysis. Analysis of NA and His was performed according to Vasanits et al. [62] using o-phthaldialdehyde and 3-mercaptopropionic acid (OPA/MPA). Ten times diluted samples were derivatized with OPA/MPA for 5 min and immediately loaded on a reversed phase column (5.0 μm, 250 mm × 4.6 mm Luna C18 (2); Phenomenex Ltd., Torrance, CA, USA) using a Shimadzu LC-20AB Prominence liquid chromatograph (Shimadzu, Kyoto, Japan). The elution gradient was as described in Vasanits et al. [62] and the flow rate was 1.2 mL min^−1^ at 40 °C. The fluorescence intensity of OPA/MPA/AA derivative was measured at excitation and emission wavelengths of 337 and 454 nm using RF-10-AXL, fluorescence detector (Prominence, Shimadzu, Kyoto, Japan). The NA and His standards were obtained from Toronto Research Chemicals (North York, ON, Canada) and Sigma-Aldrich (St. Louis, MO, USA), respectively.

### 4.5. Statistical Analysis

The data were analyzed by analysis of variance (ANOVA) and tested for significance by post hoc Tukey test at a significance level of 0.05 using SPSS 21.0 software (IBM, Armonk, NY, USA).

## 5. Conclusions

Our results indicate that both NA and His were increased under conditions of high Cu concentration in the leaves (Cu treatment), whereas only the level of His was increased when the leaf Cu accumulation declined to a moderate level (Cu + Fe treatment). However, Si addition to either Cu or Cu + Fe treatments led to an increase in concentration of both NA and His in cucumber leaves. Therefore, the results suggest that the strategy of Si-mediated alleviation of Cu toxicity was directed primarily towards the increased Cu tolerance by Cu-chelation rather than by a marked decrease of the Cu accumulation. On the other hand, the alleviation of Cu toxicity by addition of Fe was achieved through a dramatic decrease of Cu accumulation, most probably due to an antagonism between Fe and Cu. The simultaneous addition of Si and Fe showed the best ameliorative potential regarding the Cu-induced microelement deficiency, which may be of practical importance in alleviating Cu toxicity stress in plants grown on Cu contaminated soils (e.g., post-mining and post-vineyard sites).

## Figures and Tables

**Table 1:** Total plant dry weight and leaf dry weight of cucumber plants at the end of experiment. Plants were treated with excess Cu (Cu), excess Cu and Si supply (Cu + Si), excess Cu and additional Fe supply (Cu + Fe), or combined excess Cu and additional Fe and Si supply (Cu + Fe + Si); control plants (C) remained untreated.

Treatment	Dry Biomass (g Plant^−1^)	
	Total	Leaf
C	0.330 ± 0.050 c	0.223 ± 0.035 b
Cu	0.209 ± 0.047 a	0.160 ± 0.005 a
Cu + Si	0.265 ± 0.021 b	0.199 ± 0.014 b
Cu + Fe	0.291 ± 0.005 bc	0.200 ± 0.006 b
Cu + Fe + Si	0.305 ± 0.008 bc	0.217 ± 0.008 b ^1^

^1^ The values are means of four replicates ± SD. Different letters indicate significant differences among the treatments at *p* < 0.05.

**Table 2:** Total content of Cu, Fe, Zn and Mn in cucumber leaves (µmol per shoot). Plants were harvested after 5 days of treatment with excess Cu (Cu), excess Cu and Si supply (Cu + Si), excess Cu and additional Fe supply (Cu + Fe) or combined excess Cu and additional Fe and Si supply (Cu + Fe + Si); control plants (C) remained untreated.

Treatment	Microelement Content (µmol Per Shoot)			
	Cu	Fe	Zn	Mn
C	0.042 ± 0.001 a	0.460 ± 0.010 c	0.308 ± 0.027 d	0.199 ± 0.002 e
Cu	0.081 ± 0.003 c	0.168 ± 0.034 a	0.113 ± 0.008 a	0.068 ± 0.001 a
Cu + Si	0.079 ± 0.003 c	0.235 ± 0.021 ab	0.156 ± 0.020 b	0.090 ± 0.007 b
Cu + Fe	0.047 ± 0.004 ab	0.269 ± 0.008 b	0.159 ± 0.009 b	0.114 ± 0.009 c
Cu + Fe + Si	0.050 ± 0.001 b	0.401 ± 0.057 c	0.193 ± 0.017 c	0.142 ± 0.012 d ^1^

^1^ The values are means of four replicates ± SD. Different letters indicate significant differences among the treatments at *p* < 0.05.

**Table 3:** Molar ratios of Fe:Cu, Zn:Cu and Mn:Cu in cucumber leaves. Plants were harvested after 5 days of treatment with excess Cu (Cu), excess Cu and Si supply (Cu + Si), excess Cu and additional Fe supply (Cu + Fe) or combined excess Cu and additional Fe and Si supply (Cu + Fe + Si); control plants (C) remained untreated.

Treatment	Fe:Cu	Zn:Cu	Mn:Cu
C	10:1	7:1	4:1
Cu	2:1	1:1	1:1
Cu + Si	3:1	2:1	1:1
Cu + Fe	7:1	4:1	2:1
Cu + Fe + Si	8:1	4:1	3:1 ^1^

^1^ The values are means of four replicates.

**Table 4:** The molar ratios of NA:Cu, NA:Fe, NA:Zn and NA:Mn in cucumber leaves. Plants were harvested after 5 days of treatment with excess Cu (Cu), excess Cu and Si supply (Cu + Si), excess Cu and additional Fe supply (Cu + Fe) or combined excess Cu and additional Fe and Si supply (Cu + Fe + Si); control plants (C) remained untreated.

Treatment	NA:Cu	NA:Fe	NA:Zn	NA:Mn
C	3.8:1	0.4:1	0.6:1	0.9:1
Cu	2.2:1	1.1:1	1.5:1	2.6:1
Cu + Si	5.4:1	1.8:1	2.6:1	4.7:1
Cu + Fe	2.2:1	0.3:1	0.6:1	0.9:1
Cu + Fe + Si	3.4:1	0.4:1	0.8:1	1.2:1 ^1^

^1^ The values are means of four replicates.

**Table 5:** The molar ratios of His:Cu, His:Fe, His:Zn and His:Mn in cucumber leaves. Plants were harvested after 5 days of treatment with excess Cu (Cu), excess Cu and Si supply (Cu + Si), excess Cu and additional Fe supply (Cu + Fe) or combined excess Cu and additional Fe and Si supply (Cu + Fe + Si); control plants (C) remained untreated.

Treatment	His:Cu	His:Fe	His:Zn	NA:Mn
C	1.3:1	0.1:1	0.2:1	0.3:1
Cu	1.1:1	0.5:1	0.7:1	1.2:1
Cu + Si	2.2:1	0.7:1	1.1:1	1.9:1
Cu + Fe	4.6:1	0.7:1	1.2:1	1.8:1
Cu + Fe + Si	4.9:1	0.6:1	1.2:1	1.7:1 ^1^

^1^ The values are means of four replicates.

## Supplementary Materials

The following are available online at https://www.mdpi.com/2223-7747/8/12/554/s1, Figure S1: Visual appearance of the cucumber plants
