# Supplementary material for: Silicon and Iron Differently Alleviate Copper Toxicity in Cucumber Leaves
Source: Plants (Basel). 2019 Nov 28;8(12):554. doi: 10.3390/plants8120554 (PMC6963465; doi:10.3390/plants8120554)
Supplement: Supplementary file 1 [file plants-08-00554-s001.zip › Figure S1.docx]

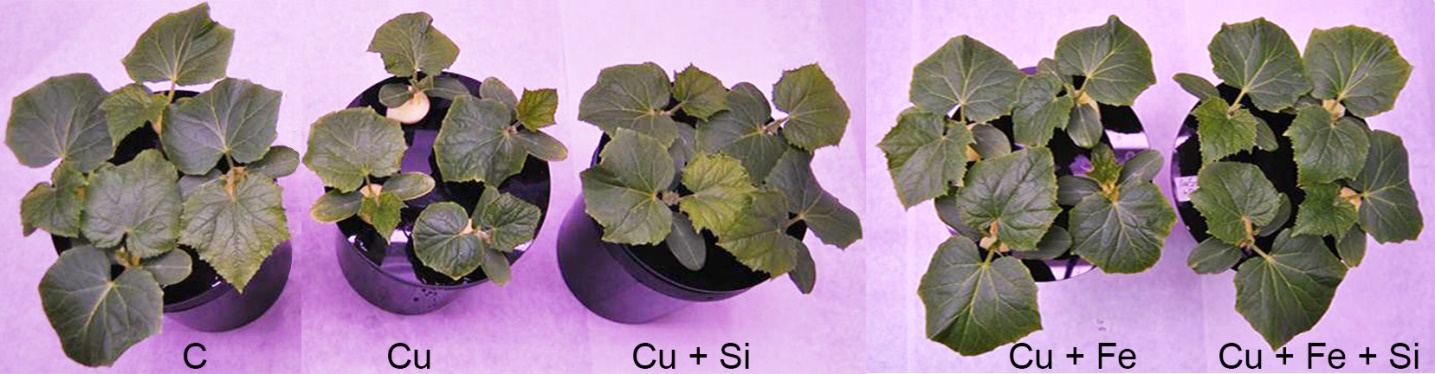


Figure S1. Visual appearance of the cucumber plants hydroponically grown for 12 days in preculture and 5 days treated with 10 μM Cu (Cu), 10 μM Cu and 1.5 mM Si (Cu+Si), 10 μM Cu and 100 μM Fe (Cu+Fe), or 10 μM Cu, 100 μM Fe and 1.5 mM Si (Cu+Fe+Si); control plants (C) remained untreated.
